# Therapeutic hypothermia in patients with coagulopathy following severe traumatic brain injury

**DOI:** 10.1186/s13049-017-0465-y

**Published:** 2017-12-20

**Authors:** Toru Hifumi, Yasuhiro Kuroda, Kenya Kawakita, Susumu Yamashita, Yasutaka Oda, Kenji Dohi, Tsuyoshi Maekawa, on behalf of the Brain Hypothermia (B-HYPO) study group in Japan

**Affiliations:** 1 Department of Emergency, Disaster and Critical Care Medicine, Kagawa University Hospital, 1750-1 Ikenobe, Miki, Kita, Kagawa, 761-0793 Japan; 2 Department of Emergency Medicine, Tokuyama Central Hospital, 1-1 Kouda, Shunan, Yamaguchi 745-8522 Japan; 3 Advanced Medical Emergency and Critical Care Center, Yamaguchi University School of Medicine, 1-1-1 Minami Kogushi, Ube, Yamaguchi 755-8505 Japan; 4 Department of Emergency Medicine, School of Medicine, Showa University, 1-5-8 Hatanodai, Shinagawaku, Tokyo, 142-8666 Japan; 5 Yamaguchi Prefectural Grand Medical Center, 77 Osaki, Boufu, Yamaguchi, 747-8511 Japan; 6 Department of Emergency, Disaster and Critical Care Medicine, 1750-1 Ikenobe, Miki, Kita, Kagawa, 761-0793 Japan

**Keywords:** Coagulopathy, Therapeutic hypothermia, Traumatic brain injury, Targeted temperature management, Fibrinogen degradation products

## Abstract

**Background:**

Coagulopathy in traumatic brain injury (TBI) has been associated with poor neurological outcomes and higher in-hospital mortality. In general principle of trauma management, hypothermia should be prevented as it directly worsens coagulopathy. Therefore, we examined the safety of mild therapeutic hypothermia (MTH) in patients with coagulopathy following severe TBI.

**Methods:**

We re-evaluated the brain hypothermia (B-HYPO) study data based on coagulopathy and compared the Glasgow Outcome Scale scores and survival rates at 6 months using per protocol analyses. Coagulopathy was defined as an activated partial thromboplastin time (APTT) > 60 s and/or fibrin/fibrinogen degradation product levels (FDP) > 90 μg/mL on admission. Baseline characteristics, coagulation parameters, and outcomes were compared between the control and MTH groups with or without coagulopathy.

**Results:**

In patients with coagulopathy, 12 patients were allocated to the control group (35.5–37.0 °C) and 20 patients to the MTH group (32–34 °C). In patients without coagulopathy, 28 were allocated to the control group and 59 patients were allocated to the MTH group.

In patients with coagulopathy, favorable neurological outcomes and survival rates were comparable between the control and MTH groups (33.3% vs. 35.0%, *P* = 1.00; 50.0% vs. 60.0%, *P* = 0.72) with no difference in complication rates. On admission, no significant differences in APTT or FDP levels were observed between the two groups; however, APTT was significantly prolonged in the MTH group compared to the control group on day 3.

**Discussion:**

Based on our study, MTH did not seem to negatively affect the outcomes in patients with coagulopathy following severe TBI on admission; therefore, the present study indicates that MTH may be applicable even in patients with severe TBI and coagulopathy.

**Conclusions:**

Our study suggests that in comparison to control, MTH does not worsen the outcome of patients with coagulopathy following severe TBI.

**Trial registration:**

UMIN-CTR, No. C000000231, Registered 13 September 2005.

**Electronic supplementary material:**

The online version of this article (10.1186/s13049-017-0465-y) contains supplementary material, which is available to authorized users.

## Background

Coagulopathy in traumatic brain injury (TBI) has been associated with poor neurological outcomes and higher in-hospital mortality [1–5]. However, in previous studies, the reported incidence of coagulopathy in isolated TBI patients has varied from 7% to 86% due to the use of differing definitions of coagulopathy [1, 6, 7].

Recently, a revised lethal triad has been proposed, with coagulopathy (fibrin/fibrinogen degradation products (FDP) levels >90 μg/mL) posited to have a central role [8]. As a general principle of trauma management, hypothermia should be prevented as it directly worsens coagulopathy. In vitro studies have demonstrated that hypothermia below 33 °C can cause coagulation dysfunction; however, the risk of bleeding associated with mild therapeutic hypothermia (MTH) is considered to be relatively small [9, 10].

Many randomized clinical trials (RCT) have been conducted to investigate the effectiveness of MTH for TBI, but they could not demonstrate more favourable outcomes than those obtained by normothermia (at 37 °C) [11–13]. However, the latest guidelines from an expert panel suggested considering TTM at 34–35 °C in order to lower ICP in TBI patients with refractory intracranial hypertension despite medical treatments [14]. Furthermore, trials examining efficacy and safety in patients with coagulopathy following TBI are yet to be conducted [11–13, 15, 16]. Therefore, we examined the hypothesis that MTH is harmful in patients with coagulopathy following severe TBI. The purpose of the present study was to examine the effect of coagulopathy on the safety of MTH compared to control in patients with severe TBI.

## Methods

### B-HYPO study

The B-HYPO study was conducted as a prospective, multicenter RCT between December 2002 and September 2008. The protocol was approved by the Institutional Review Board of each participating hospital, and the trial was registered at the University Hospital Medical Information Network site (UMIN-CTR, No. C000000231, Registered 13 September 2005) in Japan and at the National Institutes of Health site (Clinical Trials. Gov, Identifier NCT00134472, Registered 23 August 2005) in the United States of America. In brief, inclusion criteria were as follows: age 15–69 years for both sexes and a Glasgow Coma Scale (GCS) score of 4–8. Written informed consent was obtained from legally authorized representatives of patients prior to inclusion. If informed consent could not be obtained within 2 h of admission, the consent policy was waived.

### Targeted temperature management (TTM)

Treatments were performed as described in our original paper [15]. In brief, cooling was initiated within 2 h of the onset of TBI. The goal in each group was to achieve the targeted temperature within 6 h of the onset of TBI and to maintain this temperature for at least 72 h, predominantly using surface cooling blankets. After 72 h, the temperature was maintained at <38 °C until 7 days after the onset of TBI.

### Definition of coagulopathy

In previous literature, definitions of coagulopathy using the activated partial thromboplastin time (APTT) have varied from 32 s to 60 s [1, 17]. In the present study, we adopted the most severe APTT criteria (60 s). Accordingly, patients with an APTT >60 s and/or an FDP level > 90 μg/mL on admission were allocated to the coagulopathy group [18–20].

### Patients

**Fig. 1:**
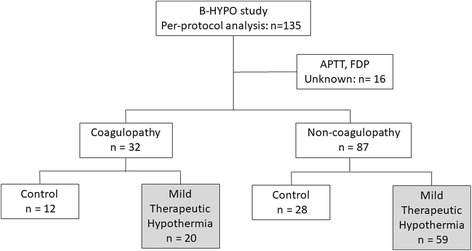
Patient flow. APTT: activated partial thromboplastin time, FDP: fibrin/fibrinogen degradation products

In the original paper, 150 patients were randomly assigned (1:2 allocation ratio) to either the control group (35.5–37.0 °C) or the MTH group (32.0–34.0 °C), and analyzed by intention to treat analyses [15]. Per-protocol analyses were performed in 135 patients (control, 47 patients and MTH, 88 patients) [21]. In the present post hoc study, we re-evaluated these data (*n* = 135) based on initial coagulation markers, APTT, and/or FDP levels. Sixteen patients (control, 7 patients and MTH, 9 patients) were excluded as either APTT or FDP values were unavailable (Fig. 1). Patients were classified as either coagulopathy (*n* = 32, 26.9%) or non-coagulopathy (*n* = 87, 73.1%). In patients with coagulopathy, 12 patients were allocated to the control group and 20 patients to the MTH group. In patients without coagulopathy, 28 were allocated to the control group and 59 patients were allocated to the MTH group.

### Data collection and study outcomes

Data on the following parameters were collected: age, gender, systolic blood pressure, heart rate, GCS, unreactive pupil or pupils, platelet counts, APTT, fibrinogen, FDP, Traumatic Coma Data Bank classification, Injury Severity Score (ISS), Abbreviated ISS (AIS) for the head, AIS score ≥ 4 for other organs on admission, complication rate during TTM, surgical intervention for TBI during administration, and favorable neurological outcomes and survival rates at 6 months following TBI. APTT and FDP levels in the acute phase (time to admission to day 3) were compared between the control and MTH groups with or without coagulopathy, respectively. Platelet counts in the acute phase (time to admission to day 3) were also compared between the control and MTH groups with coagulopathy.

The primary outcomes were favorable neurological outcomes, survival rates, and complication rates between coagulopathy and non-coagulopathy patients, and between the control and MTH groups with or without coagulopathy. The Glasgow Outcome Scale (GOS) at 6 months, was assessed by a neurosurgeon, a neurologist, or an emergency physician who was unaware of the patient’s treatment. Good recovery and moderate disability according to the GOS scores at 6 months after injury were designated as a favorable neurological outcome. Fatal arrhythmia, thrombocytopenia, severe pneumonia, sepsis, and disseminated intravascular coagulation were defined as complications [22].

### Statistical analyses

Baseline characteristics were compared between coagulopathy and the non-coagulopathy patients. Then, baseline characteristics, favorable neurological outcomes, and survival and complication rates were compared between the control (35.5–37 °C) and MTH (32–34 °C) groups, with or without coagulopathy. Changes in APTT and FDP levels from admission to day 3 and ICP from day 1 to 1 day after rewarming were compared between the control and MTH groups with or without coagulopathy.

Continuous variables were analyzed using the Mann–Whitney U test and categorical comparisons were performed using Fisher’s exact test. Statistical analyses were performed using JMP version 11 software (SAS Institute, Cary, NC, USA). Results are presented as n (%) or medians (interquartile ranges, IQRs). *P*-values ≤0.05 were considered statistically significant.

## Results

### Comparison of baseline characteristics, neurological outcomes, survival rates, and complication rates between coagulopathy and non-coagulopathy patients

Coagulopathy occurred in 26.9% of included patients. No significant differences in baseline characteristics were observed between coagulopathy patients and non-coagulopathy patients, except for gender (Table 1). As expected, APTT in coagulopathy patients was significantly prolonged when compared to non-coagulopathy patients (median, IQR: 42.9 s [34.2–84.5] vs. 28.3 s [25.5–34.3], *P* < 0.01). The initial FDP in coagulopathy patients was significantly higher than that in non-coagulopathy patients (114.8 μg/mL [92.5–168.3] vs. 30.9 μg/mL [14.2–50.3], *P* < 0.01).

**Table 1:** Patient characteristics

	Coagulopathy *n* = 32	Non-coagulopathy *n* = 87	*P*-value
Age (years)	42 (22–56)	42 (21–55)	0.55
Male	27 (87.1)	52 (61.1)	< 0.01
Systolic blood pressure (mmHg)	151 (124–170)	142 (114–179)	0.48
Heart rate (beats/min)	83 (71–110)	86 (70–106)	0.89
Glasgow Coma Scale score	6 (4–7)	6 (4–7)	0.60
Unreactive pupil or pupils	14 (43.8)	37 (42.5)	1.00
Platelet counts (×10^4^/mm^3^)	23.7 (18.4–29.3)	22.7 (17.1–27.4)	0.52
APTT (s)	42.9 (34.2–84.5)	28.3 (25.5–34.3)	< 0.01
FDP (μg/mL)	114.8 (92.5–168.3)	30.9 (14.2–50.3)	< 0.01
Fibrinogen (mg/dL)	206 (140–267)	202 (165–243)	0.87
TCDB classification			0.77
Diffuse injury grade I	0 (0)	2 (2.3)	
Diffuse injury grade II	11 (34.4)	25 (28.7)	
Diffuse injury grade III	7 (21.9)	12 (13.8)	
Diffuse injury grade IV	1 (3.1)	3 (3.5)	
Non-evacuated mass/Evacuated mass	1/12	4/41	0.68
Surgical operation for TBI	16 (51.6)	58 (66.7)	0.19
Injury severity score	25 (21–34)	25 (17–30)	0.09
AIS score for head	4 (4–5)	4 (4–5)	0.16
Favorable outcome	11 (34.4)	44 (50.6)	0.15
Survival rate	18 (56.3)	63 (72.4)	0.12
Overall complication rate	4 (12.5)	12 (13.8)	0.85

Values are presented as n (%) or median (interquartile ranges, IQRs)

*MTH* mild therapeutic hypothermia, *AIS* abbreviated injury score, *TBI* traumatic brain injury, *CT* computed tomography, *FDP* fibrin degradation products, *TCDB* Traumatic Coma Data Bank

Although no significant difference in favorable neurological outcome (34.4% vs. 50.6%, *P* = 0.15) and survival rates (56.3% vs. 72.4%, *P* = 0.12) was observed between coagulopathy and non-coagulopathy patients, these values were lower in coagulopathy patients. There was no difference between two groups in the complication rate (*P* = 0.85).

### Comparison of baseline characteristics between the control (35.5–37 °C) and MTH (32–34 °C) groups in the patients with or without coagulopathy

No significant differences in patient characteristics were observed between the control and MTH groups among patients with or without coagulopathy (Table 2).

**Table 2:** Comparison of patient characteristics

Variable	Coagulopathy			Non-Coagulopathy		
	Control (35.5–37.0 °C) *n* = 12	MTH (32.0–34.0 °C) *n* = 20	*P*-value	Control (35.5–37.0 °C) *n* = 28	MTH (32.0–34.0 °C) *n* = 59	*P*-value
Age (years)	31 (21–55)	48 (25–58)	0.34	41 (23–57)	42 (20–55)	0.93
Male	9 (81.8)	18 (90.0)	0.60	16 (59.3)	36 (62.1)	0.82
Systolic blood pressure (mmHg)	162 (128–177)	138 (122–160)	0.15	142 (115–183)	143 (110–175)	0.88
Heart rate (beats/min)	77 (71–95)	94 (71–113)	0.34	86 (65–106)	85 (72–104)	0.94
Glasgow Coma Scale score	6 (5–7)	6 (4–7)	0.72	6 (5–7)	6 (4–7)	0.65
Unreactive pupil or pupils	5 (41.7)	9 (45.0)	1.00	12 (42.9)	25 (42.4)	1.00
Platelet counts (×10^4^/mm^3^)	22.4 (18.7–29.3)	24.5 (16.8–29.2)	0.91	26.2 (16.7–29.8)	22.1 (17.1–26.2)	0.14
APTT (s)	42.8 (27.3–81.9)	44.9 (35.4–87.1)	0.73	27.4 (24.3–36.2)	28.9 (25.7–33.2)	0.58
FDP (μg/mL)	168 (88.1–200.5)	106 (92.4–142.2)	0.17	37.5 (12.3–47.9)	26 (16.5–55.6)	0.91
Fibrinogen (mg/dL)	228 (125–271)	189 (164–246)	0.94	203 (167–264)	199 (164–235)	0.49
TCDB classification			0.77			0.30
Diffuse injury grade I	0 (0)	0 (0)		1 (3.6)	1 (1.7)	
Diffuse injury grade II	5 (41.7)	6 (30.0)		8 (28.6)	17 (28.8)	
Diffuse injury grade III	2 (16.7)	5 (25.0)		6 (21.4)	6 (10.2)	
Diffuse injury grade IV	0 (0)	1 (5.0)		2 (7.1)	1 (1.7)	
Non-evacuated mass/Evacuated mass	0/5	1/7	0.81	0/11	4/30	0.56
Surgical operation for TBI	7 (58.3)	9 (47.4)	0.72	18 (64.3)	40 (67.8)	0.81
Injury severity score	25 (21–25)	34 (22–36)	0.08	22 (16–29)	25 (17–34)	0.36
AIS score for head	5 (4–5)	4 (4–5)	0.50	4 (4–5)	4(4–5)	0.20
AIS score ≥ 4 for other organs	0 (0)	4 (20.0)	0.27	3 (10.7)	4 (6.8)	0.67

Values are presented as n (%) or median (interquartile ranges, IQRs)

*MTH* mild therapeutic hypothermia, *AIS* abbreviated injury score, *TBI* traumatic brain injury, *CT* computed tomography, *FDP* fibrin degradation products, *TCDB* Traumatic Coma Data Bank

### Comparison of neurological outcomes, survival rates and complication rates between the control and MTH groups in patients with or without coagulopathy

Among patients with coagulopathy, favorable neurological outcomes and survival rates were comparable between the control and MTH groups (33.3% vs. 35.0%, *P* = 1.00; 50.0% vs. 60.0%, *P* = 0.72) with no difference in complication rates (Table 3, left).

**Table 3:** Comparison of neurological outcomes and complication rates between coagulopathic and non-coagulopathic patients, and between the MTH (32–34 °C) and control (35.5–37 °C) groups with or without coagulopathy

Variable	Coagulopathy *n* = 32			Non-Coagulopathy *n* = 87		
	Control (35.5–37.0 °C) *n* = 12	MTH (32.0–34.0 °C) *n* = 20	*P*-value	Control (35.5–37.0 °C) *n* = 28	MTH (32.0–34.0 °C) *n* = 59	*P*-value
Favorable outcome	4 (33.3)	7 (35.0)	1.00	16 (57.1)	28 (47.5)	0.49
Survival rate	6 (50.0)	12 (60.0)	0.72	25 (89.3)	38 (64.4)	0.02
Overall complication rate	1 (8.3)	3 (15.0)	1.00	0 (0)	12 (20.3)	< 0.01

*MTH* mild therapeutic hypothermia

Neurological outcomes were evaluated 6 months after brain injury

Complications occurring during targeted temperature management were recorded

In patients without coagulopathy, the survival rate was significantly lower and the complication rate was significantly higher in the MTH group compared to the control group (89.3% vs. 64.4%, *P* = 0.02 and 0% vs. 20.3%, *P* < 0.01, respectively; Table 3, right).

### Comparison of APTT and FDP levels between the fever control and MTH groups in patients with or without coagulopathy

**Fig. 2:**
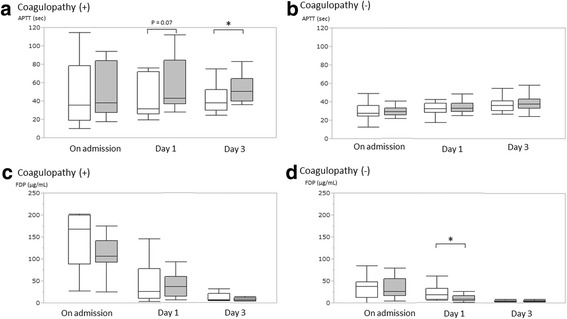
Alterations of APTT and FDP between the fever control and MTH groups in patients with or without coagulopathy. **a** Comparisons of APTT between the control and the MTH groups in the patients with coagulopathy. **b** Comparisons of APTT between the control and the MTH groups in the patients without coagulopathy. **c** Comparisons of FDP between the control and the MTH groups in the patients with coagulopathy. **d** Comparisons of FDP between the control and the MTH groups in the patients without coagulopathy. The control group (35·5 °C–37 °C) is indicated in white and the MTH group (32 °C–34 °C) is indicated in gray. The boxes are the 25th to 75th percentiles and the whiskers are 5th to 95th percentiles. *p < 0.05; statistically significant. APTT: activated partial thromboplastin time, FDP: fibrin/fibrinogen degradation products

In patients with coagulopathy, there was no significant difference in APTT or FDP levels between the two groups at the time of admission; however, APTT was significantly prolonged in the MTH group compared to the control group on day 3 (*P* < 0.05; Fig. 2).

In patients without coagulopathy, there was no significant difference in APTT or FDP levels between the two groups at the time of admission; however, FDP levels were significantly lower in the MTH group compared to the control group on day 1 (*P* < 0.05; Fig. 2).

### Comparison of platelet counts between the control and MTH groups in patients with coagulopathy

In patients with coagulopathy, there were no significant differences in platelet counts between the two groups at the time of admission to 1 day after rewarming (Additional file 1).

### Comparison of ICP between the fever control and MTH groups in patients with or without coagulopathy

In patients with coagulopathy, the median ICP was 20 (10–46) mmHg, 21 (14–34) mmHg, and 19 (13–37) mmHg on day 1, day 3, and 1 day after rewarming, respectively. In patients without coagulopathy, the median ICP was 15 (10–22) mmHg, 15 (9–19) mmHg, and 21 (16–33) mmHg on day 1, day 3, and 1 day after rewarming, respectively. ICP was significantly higher in patients with coagulopathy compared to patients without coagulopathy on day 3 (*p* < 0.01).

**Fig. 3:**
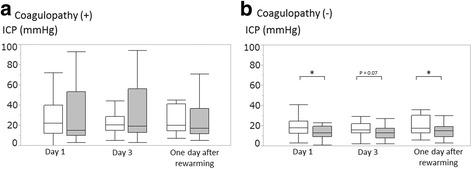
Alterations of ICP between the control and MTH groups in patients with or without coagulopathy*.* **a** Comparisons of ICP between the control and the MTH groups in the patients with coagulopathy. **b** Comparisons of ICP between the control and the MTH groups in the patients without coagulopathy. The control (35·5–37 °C) group is indicated in white and the MTH (32–34 °C) group is indicated in gray. The boxes are the 25th to 75th percentiles and the whiskers are 5th to 95th percentiles. *p < 0.05; statistically significant

In patients with coagulopathy, ICP did not differ between the control and MTH groups on day 1, day 3, or 1 day after rewarming (Fig. 3, left). In patients without coagulopathy, ICP was significantly lower in the MTH group compared to the control group on day 1 and at 1 day after rewarming (Fig. 3, right).

## Discussion

In the present post hoc study, coagulopathy occurred in 32 (26.9%) of 119 patients with severe TBI. In patients with coagulopathy, favorable neurological outcomes were recorded in one third of patients and their survival rate was greater than 50%. Outcomes were similar between the MTH and control groups with no significant difference in complication rate, although prolongation of APTT lasted to day 3 in the MTH group. Consequently, we posit that both control and MTH have utility in the treatment of severe TBI in patients with coagulopathy on admission.

Tokutomi et al. examined the effects of hypothermia on several coagulation parameters (PT, APTT, platelet count, and AT-III) and performed a comparison with normothermia in TBI patients. They reported a trend toward a prolonged APTT in the hypothermia group on days 5 and 7 (*P* = 0.07 and *P* = 0.06, respectively). In the present study, the APTT in the MTH group at the time of admission (28.8 ± 2.7 s) did not differ from that of the control group (29.3 ± 5.7 s). Although the inclusion criterion used in the present study was a severely prolonged APTT of >60 s [23], the APTT in the MTH group among patients with coagulopathy was comparable to Tokutomi’s data and found to be significantly prolonged on day 3 compared to the control group [23].

Genet et al. examined the pathophysiology of trauma-induced coagulopathy between isolated TBI and non-TBI patients, and concluded that hemostatic, vascular, and endothelial responses were comparable [24]. Theoretically, APTT indicates the activity of the intrinsic and common pathways of coagulation, and prolongation of the APTT indicates the presence of a coagulation disorder [25]. Among patients with coagulopathy included in the present study, APTT was prolonged in the MTH group compared to the fever control group and reached statistical significance on day 3 (Fig. 2a). These findings are attributable to the speed of decreased biochemical reactions due to the decreased core body temperature in the MTH group. A similar trend was observed in patients without coagulopathy; however, this difference did not reach statistical significance (Fig. 2b). On the other hand, FDP levels in patients with coagulopathy were markedly elevated at the time of admission, as expected (Fig. 2c). The elevation indicated acceleration of systemic fibrin deposition and secondary fibrinolysis/fibrinogenolysis by plasmin [24]. The values gradually decreased until day 3, both in the control and MTH groups, without statistical significance.

Polderman reported that very mild hypothermia (35 °C) does not affect coagulation and can be safely used even in patients at high risk of bleeding [9]. In addition, regarding mild hypothermia (33–35 °C), Wolberg et al. examined healthy volunteers and found that enzyme activities and platelet activation were not reduced at 33 °C [26]. Therefore, Gando et al. posited that isolated mild hypothermia at 33 to 35 °C does not have severe effects on hemostasis in typical clinical trauma settings [27]. Based on our study, MTH did not seem to negatively affect the outcomes in patients with coagulopathy following severe TBI on admission; therefore, the present study indicates that MTH may be applicable even in patients with severe TBI and coagulopathy. Typically, TBI-associated coagulopathy is not related to visual blood loss [28]; therefore, clinicians should attend to intracranial hemorrhage and organs with ongoing bleeding. In fact, patients with an AIS score ≥ 3 for other organs treated with mild therapeutic hypothermia had a mortality rate greater than 80% in the present study (data not shown).

In the patients with coagulopathy, ICP tended to be high with wide ranges both in the control and the MTH groups, compared to those in the patients without coagulopathy during the periods of the targeted temperature managements, day 1 and day 3 (Fig. 3a, b). These differences might contribute their low rates of favorable neurological outcome and survival both in the fever control and MTH groups with coagulopathy (Table 3).

There are several limitations to the present study. First, the original study was terminated before the full sample size had been recruited. Additionally, the sample size was further reduced from 150 to 119 patients as APTT and/or FDP values could not be obtained in 16 out of 135 patients. These factors may have biased the outcomes of the present study. Second, d-dimer levels are the most specific test for coagulopathy in TBI [29]; however, the number of patients with d-dimer levels measured at the time of arrival was small in the present study. Further, PT could not be examined due to unavailability of the dataset. Third, an extremely small number of patients were included in this study (coagulopathy occurred in only 32 patients), and the results require confirmation in a larger cohort. Beta-error may have also existed. Fourth, although there was no significant difference in age (*p* = 0.34), patients in fever control group were younger than those in MTH group (median age 31 years vs. 48 years). Therefore, additional studies adjusted for background factors will be required. Finally, as the present study was a post hoc sub-analysis, selection bias may have been present.

## Conclusion

Our study suggests that in comparison to control, MTH does not worsen the outcome of patients with coagulopathy following severe TBI.

## Additional file

Additional file 1: Comparison of platelet counts between the control and MTH groups in patients with coagulopathy. MTH, mild therapeutic hypothermia. Values are presented as median (interquartile ranges, IQRs). (DOCX 13 kb)

## Abbreviations

AIS: Abbreviated Injury Severity Score
APTT: activated partial thromboplastin time
B-HYPO: Brain Hypothermia study group
FDP: fibrin/fibrinogen degradation products
GCS: Glasgow Coma Scale
ICP: intracranial pressure
ISS: Injury Severity Score
MTH: mild therapeutic hypothermia
RCT: randomized controlled trial
TBI: traumatic brain injury
TTM: targeted temperature management
