# Supplementary material for: Therapeutic hypothermia in patients with coagulopathy following severe traumatic brain injury
Source: Scand J Trauma Resusc Emerg Med. 2017 Dec 20;25:120. doi: 10.1186/s13049-017-0465-y (PMC5738813; doi:10.1186/s13049-017-0465-y)
Supplement: Additional file 1: — Comparison of platelet counts between the control and MTH groups in patients with coagulopathy. MTH, mild therapeutic hypothermia. Values are presented as median (interquartile ranges, IQRs). (DOCX 13 kb) [file 13049_2017_465_MOESM1_ESM.docx]

**Additional file 1**

**Comparison of platelet counts between the control and MTH groups in patients with coagulopathy**

|  | MTH (×10^4^/mm^3^) | Control (×10^4^/mm^3^) | P value |
| --- | --- | --- | --- |
| Day 0 | 23.7 (18.4-29.3) | 22.7 (17.1-27.4) | 0.62 |
| Day 1 | 12.4 (8.3-16) | 12.5 (9.7-12.5) | 0.52 |
| Day 3 | 8.1 (6.1-11.8) | 8.6 (6.8-12.7) | 0.28 |
| One day after rewarming | 10.4 (7.3-19.7) | 11.4 (8.0-15.1) | 0.98 |

MTH, mild therapeutic hypothermia.

Values are presented as median (interquartile ranges, IQRs).
